# Changes in stability and shelf-life of ultra-high temperature treated milk during long term storage at different temperatures

**DOI:** 10.1016/j.heliyon.2019.e02431

**Published:** 2019-09-12

**Authors:** Maria A. Karlsson, Maud Langton, Fredrik Innings, Bozena Malmgren, Annika Höjer, Malin Wikström, Åse Lundh

**Affiliations:** a Swedish University of Agricultural Sciences, Department of Molecular Sciences, P.O. 7015, 75007, Uppsala, Sweden; b Tetra Pak Processing Systems AB, Ruben Rausings gata, 22186, Lund, Sweden; c Norrmejerier Ek. Förening, Mejerivägen 2, 90622, Umeå, Sweden

**Keywords:** Food technology, Food safety, UHT milk, Sensory evaluation, Off-flavour, Sedimentation, Colour, Food acceptance, Food quality, Food science, Food storage, Shelf life of foods

## Abstract

In the ultra-high temperature (UHT) process, milk is subject to temperatures above 135 °C for few seconds giving a product with a shelf-life of several months. The raw milk quality, UHT process and storage conditions affect the stability. In this study, the stability of UHT milk produced in an indirect system was evaluated by studying changes in taste, colour, fat separation, fat adhesion to the package, sedimentation, gelation, heat coagulation time, pH and ethanol stability during storage for up to one year at different temperatures. UHT milk stored at 4 and 20 °C had the longest shelf-life of 34–36 weeks, limited by sediment formation. Storage at 30 and 37 °C considerably decreased the shelf-life of UHT milk to 16–20 weeks, whereby changes in sediment formation, taste and colour were the limiting factors. Our results suggest that the changes observed at the different storage temperatures can be explained by different known mechanisms.

## 1 Introduction

In the ultra-high temperature (UHT) process, milk is subjected to high temperatures, above 135 °C for a few seconds, resulting in a product with a shelf-life of several months when stored at ambient temperature. Chemical and physical changes in the milk can lead to off-flavours, browning, fat separation, sediment formation or gelation during the subsequent storage (Anema, 2017; Deeth, 2010). Important factors affecting the changes of UHT milk are processing parameters, storage conditions (time and temperature) and type of packaging (Deeth, 2010; Schiano et al., 2017).

From a consumer perspective, the sensory attributes of the milk including taste and colour as well as visual stability such as fat separation, fat adhesion to the packaging material and sediment formation are of significant importance. Sensory methodologies to assess dairy products range from simple tests with untrained persons to trained panels, consumer surveys and chemical analysis (Schiano et al., 2017). The sensory perception and appreciation of milk varies greatly among consumers (Walstra et al., 2006) and in milk is mainly affected by the fat, protein and lactose content, as well as the manufacturing process (Oupadissakoon et al., 2009; Schiano et al., 2017). Compared to pasteurized milk, UHT milk has due to the severe heating, more of cooked and sulphuric flavours (Schiano et al., 2017). These flavours are generally regarded as undesirable by Swedish consumers, hence, this is one of the reasons why UHT milk only has a limited market share in Sweden. Upon long-term storage of UHT milk, the main processes suggested to affect the taste and colour are proteolytic, lipolytic, oxidative and Maillard reaction, initiated by the intense heat treatment of UHT milk (van Boekel, 1998; Walstra et al., 2006). In the initial stages of the Maillard reaction, the reducing sugar in milk (mainly lactose) condenses with amino groups (mainly lysine residues) and the so-called Amadori product, in milk protein bound lactulosyllysine, is formed (van Boekel, 1998). Volatile compounds are mainly formed in the intermediate stage (Sunds et al., 2018) and in the final stage of the Maillard reaction, a non-enzymatic browning reaction consisting of condensation of amino compounds and sugar fragments, leads to formation of polymerized protein and brown melanoidins (Al-Saadi and Deeth, 2008; van Boekel, 1998). In a previous study by Hansen et al. (1980), an untrained panel tested UHT milk heated to 149 °C for 3.4 s and found the taste to slightly improve when the milk was stored refrigerated at 4 °C for 24 weeks compared to storage at 40 °C. In a study by Fink and Kessler (1986), UHT milk was stored at 4, 20 and 35 °C and an organoleptic test was performed by a trained panel. It was reported that the taste of the UHT milk deteriorated slightly with increased storage temperature, but it was still considered acceptable at 20 weeks of storage.

During storage of UHT milk, fat globules can aggregate and float to the top, resulting in fat separation and fat adhesion to the packaging material (Ramsey and Swartzel, 1984). Fat separation is closely correlated to and will increase with fat content, storage temperature and fat globule size (Hardham et al., 2000; Lu et al., 2013). The rate of fat separation is affected by the homogenisation efficiency in which a higher efficiency retards the fat separation by contributing to a larger reduction in fat globule size (Lu et al., 2013). In UHT products, sediment formation and gelation are also well-known problems. Sediment, forming a compact layer adhering at the bottom of the package, is suggested to consists of aggregates of proteins or protein particles of various sizes (Gaur et al., 2018; Malmgren et al., 2017). Sediment formation has been shown to increase with storage temperature (Gaur et al., 2018; Malmgren et al., 2017; Ramsey and Swartzel, 1984). In contrast, gelation, consisting of a three-dimensional, voluminous network of proteins, can occur either through enzymatic or non-enzymatic (i.e. physio-chemical) processes (Anema, 2017).

Various properties of the unprocessed milk are being used for quality assurance of the raw milk used for UHT treatment. A commonly applied method to predict the heat stability of milk is determination of the heat coagulation time (HCT), i.e. the time it takes for milk to visually coagulate when heated to temperatures above 100 °C (Davies and White, 1966). Earlier studies have measured HCT of the raw milk, i.e. before UHT processing, and found that milk with low HCT is associated to increased fouling in UHT processing and sediment formation during storage (Deeth and Lewis, 2016). The HCT primarily depends on the initial pH, calcium activity and casein micelle size (van Boekel et al., 1989) and even small changes in pH have been found to result in major changes in heat stability (Deeth and Lewis, 2016; Jeurnink and de Kruif, 1995; van Boekel et al., 1989). Milk has a natural pH around 6.7, and lowering the pH will reduce the negative net charge of proteins, thereby promoting protein-protein interactions (Day et al., 2017). According to manufacturers of UHT processing equipment, raw milk should have a pH above 6.65 to not cause UHT processing problems, e.g. fouling on the heating surfaces (Tetra Pak, 2018). Ethanol stability has been used for over a century as a simple, cheap and quick pass-fail-test to detect low quality raw milk that is not suitable for UHT processing (Horne, 2016). It has been recommended that raw milk for UHT processing should have an ethanol stability of 74% or higher (Shew, 1981). Reasons suggested to explain low ethanol stability of the raw milk include low pH caused by acid producing bacteria, salt imbalance, high concentration of ionic calcium or high amount of serum proteins (colostrum) (Horne, 2016; Lewis et al., 2011). In addition to the application of HCT and ethanol stability to assess the suitability of raw milk for UHT processing, there are only few studies reporting their use in evaluating changes in UHT milk stability during storage (Gaucher et al., 2008a,b; Samel et al., 1971).

The aim of this study was to investigate the shelf-life of UHT milk by frequent sampling from a commercial and a small pilot scale production site, followed by long-term storage, up to one year after production, to identify the sensory changes limiting the shelf-life of the product when stored at different temperatures. For this purpose quality traits including taste, colour, fat separation, fat adhesion, sedimentation and gelation were evaluated. In addition, changes in HCT, pH and ethanol stability of the UHT milk were evaluated and used to suggest explanations behind the observed changes.

## 2 Materials and methods

### 2.1 Sample preparation, handling and storage

UHT milk was produced at two sites in Sweden, i.e. at a commercial dairy plant in Luleå (Norrmejerier) and in a pilot scale facility in Lund (Tetra Pak Product Development Centre). From the dairy plant, in total eleven batches of UHT milk were collected on a monthly basis during one year, with the exception of August, whereas from the pilot plant, two batches were produced; one in November and another one in February. An earlier published study, describing the properties of the commercial raw milk and the corresponding freshly produced UHT milk provides detailed information on the milk composition, including total bacterial count, somatic cell count and enzymatic activity (Karlsson et al., 2017). In the unprocessed commercial raw milk collected during the indoor period, we observed on average total bacterial counts of 29′000 cfu/mL, somatic cell count of 182′000 cells/mL and total proteolysis of 40.58 mM leucine equivalents. Only minor variations in raw milk composition and the resulting, freshly produced UHT milk were found. Therefore, in this study the eleven batches of UHT milk were treated as replicates in the statistical evaluation.

At the dairy raw milk was used and at the pilot plant pasteurised milk was used for further processing. At both sites, the milk was standardized (1.5% fat), upstream two-stage homogenised (150 + 30 bar) and UHT treated using indirect tubular heat exchangers at 137 °C for 4 s. The UHT milk was aseptically packed in 1 L (dairy plant) or 250 mL (pilot plant) Tetra Brik Aseptic (TBA) packages and transported to the Swedish University of Agricultural Sciences (SLU), Uppsala, at ambient temperature. The UHT milk was analysed the day after arrival to SLU, giving UHT milk with an age of 1–2 weeks after production, and thereafter it was stored at 4, 20, 30 and 37 °C for up to 52 weeks age. During storage, every fourth week, new TBA packages with milk were opened and analysed. Before the evaluation, samples were stored at room temperature overnight and analysed the following morning. All measurements were done at ambient temperature unless else stated.

### 2.2 Taste, colour, fat separation, fat adhesion, sediment formation and gelation

**Fig. 1:**
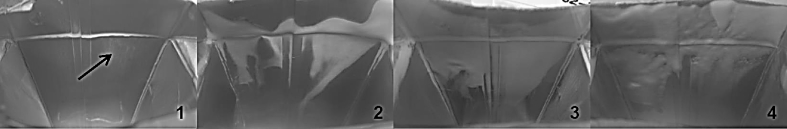
Examples of reference photos to estimate fat adhesion, i.e. the area and thickness of the fat layer adhering to the inside of the package after the milk was poured out (see arrow), graded on a scale 0–4.

**Fig. 2:**
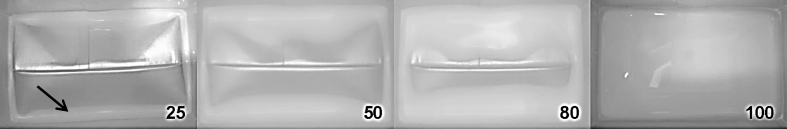
Examples of reference photos to estimate the amount of sediment formed at the bottom of the package (see arrow), here corresponding to 25, 50, 80 and 100% sediment. Sediment formation of 0% corresponded to no sediment and at 100% the bottom of the package was completely covered by sediment.

Sensory evaluation, including tasting of the milk, was performed by a trained person from storage week 16 to 52 for UHT milk produced at the dairy plant in January, March, May, July, September and November. The taste was graded as normal taste, small taste deviation or large deviation and, if possible, the deviating taste was described by words. The CIELAB colour space was measured with a CM-600d spectrophotometer (Konica Minolta, Shanghai, China). Using this technology, L* indicates lightness ranging from 0-100, a* indicates a range from green to red (-60 to +60) and b* a range from blue to yellow (-60 to +60). To evaluate the fat separation, defined as the thickness of the cream layer floating on the surface, the flaps on the packages were turned up, the top of the package cut off and the thickness of the cream layer was rated on a four graded scale; no visual cream layer, waves of cream, a surface completely covered with fat or lumps/clots of fat. Fat adhesion, defined as the thickness and amount of fat adhering to the inside of the package after the milk was poured out, was compared with reference photos and rated on a scale 0–4 (Fig. 1), modified from a protocol of New Zealand Dairy Industry (2000a). The amount of sediment formed at the bottom of the package was visually estimated and compared with reference photos, and graded on a scale 0–100%, where 0 corresponded to no sediment and 100 corresponded to the bottom of the package completely covered by sediment (Fig. 2) (New Zealand Dairy Industry, 2000b). Samples were visually inspected for absence or presence of age gelation.

### 2.3 Heat coagulation time, pH and ethanol stability

HCT, pH and ethanol stability of UHT milk was measured as described in Karlsson et al. (2017). In brief, HCT was defined as the time needed for visual coagulation of 0.5 mL milk in a sealed test tube whilst being rocked at 130 °C (Davies and White, 1966) using the dedicated equipment from Hettich Benelux (Geldermalsen, Netherlands). The pH was measured using an IoLine electrode (SI Analytics®, Mainz, Germany). Ethanol stability was defined as the highest ethanol concentration that could be added to the sample without causing visual coagulation of the milk. Equal volumes of milk and ethanol, at ethanol concentrations ranging between 40 and 100% in 2% increments, were mixed in an Eppendorf tube and incubated for 30 min before reading (White and Davies, 1958).

### 2.4 Statistical analysis

Minitab 18 software (Minitab Ltd, State College, Pennsylvania) was used to calculate Pearson's correlation coefficients. P-values were calculated by one-way ANOVA (two-sided 95% confidence interval).

## 3 Results

### 3.1 Taste, colour, fat separation, fat adhesion, sediment formation and gelation

From storage week 16, every second batch of UHT milk produced at the dairy plant was subject to a sensory evaluation. For the UHT milk stored at 4 and 20 °C, a deviating taste was in general observed from storage weeks 48–52 and 40–44, respectively. For UHT milk stored at 30 and 37 °C a deviating taste was generally detected from storage weeks 24–32. For all storage temperatures, the deviating taste was described as sweet, cardboard, creamy or watery. Additionally, for UHT milk stored at 30 and 37 °C, the deviating taste was described as acidic or caramel.

**Fig. 3:**
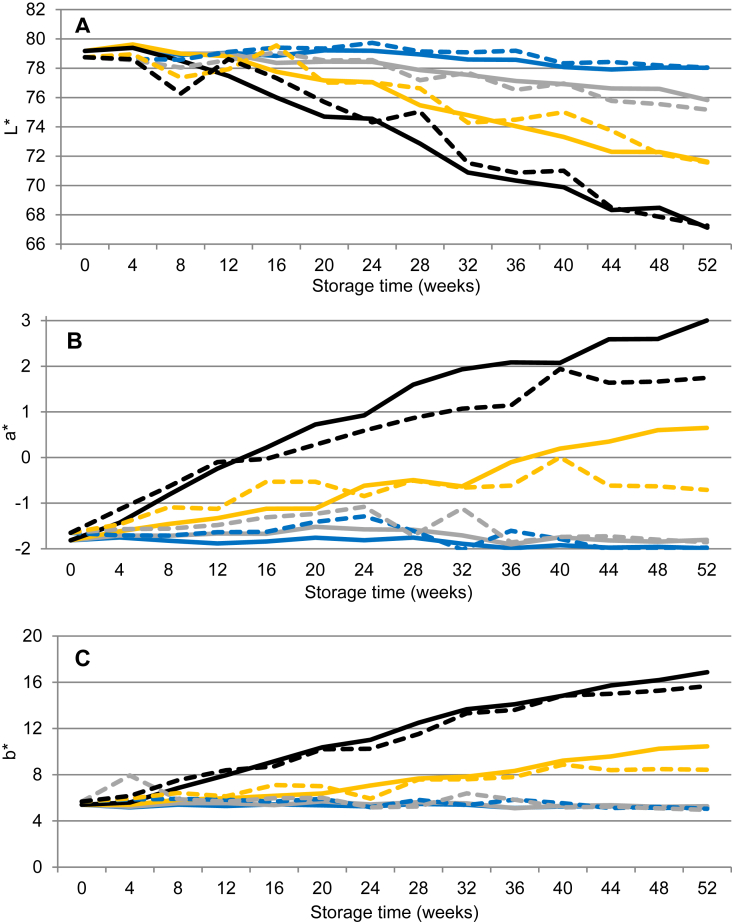
Changes in L* (dark-light), a* (green-red) and b* (blue-yellow) in ultra-high temperature treated milk during storage from 0-52 weeks at 4 °C (blue), 20 °C (grey), 30 °C (yellow) and 37 °C (black). Values represent average values of eleven batches of UHT milk produced at a dairy plant (solid line) and two batches of UHT milk produced at a pilot plant (dashed line).

Throughout the long-term storage of 52 weeks, the lightness (L*) of UHT milk stored at 4 °C remained >78, whereas for UHT milk stored at 20, 30 and 37 °C, L* values decreased linearly to 75–76, 72 and 67, respectively (Fig. 3A). In UHT milk stored at 4 and 20 °C, a* and b* values did not change during storage. The UHT milk stored at 37 °C became considerably more red and yellow, corresponding to a* and b* values increasing from -2 to 3 and from 6 to 16 (Figs. 3B and 3C), respectively. The milk had a brown colour from storage weeks 28–30 and 16–20 when stored at 30 and 37 °C, respectively. This unacceptable colour typically corresponded to L* values < 76, a* values > -1 and b* values > 7.

**Fig. 4:**
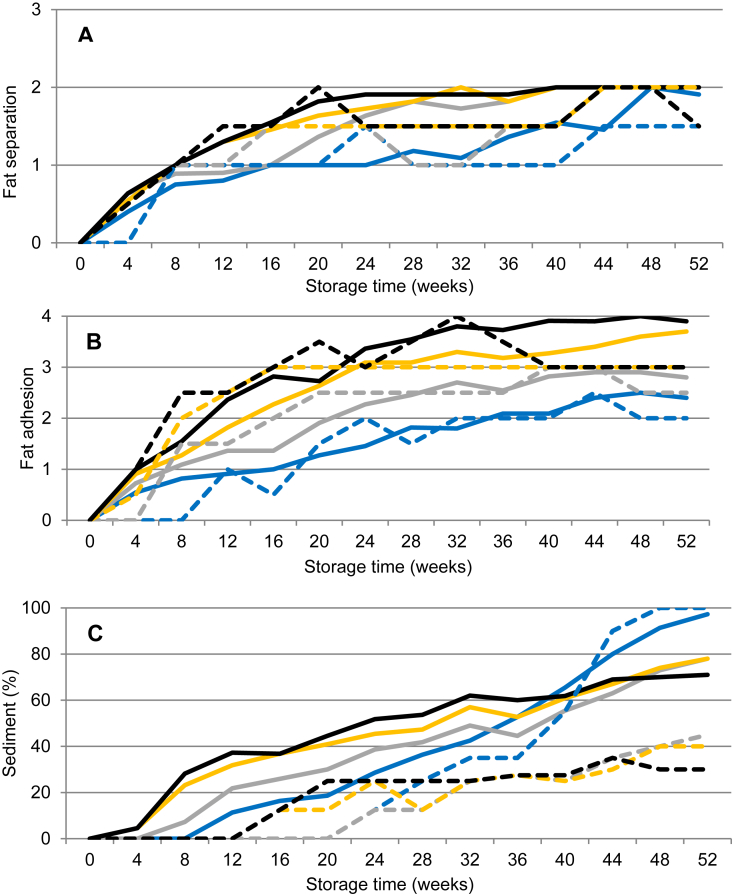
Changes in fat separation, fat adhesion and sediment formation in ultra-high temperature treated milk during storage from 0-52 weeks at 4 °C (blue), 20 °C (grey), 30 °C (yellow) and 37 °C (black). Values represent average values of eleven batches of UHT milk produced at a dairy plant (solid line) and two batches of UHT milk produced at a pilot plant (dashed line).

An initial fat separation was observed for all milks during the first 8–16 weeks of storage (Fig. 4A). After approximately 40 weeks of storage for UHT milk stored at 4 °C, and already after 20 weeks when stored at 37 °C, fat separation increased from waves of cream to a surface completely covered with fat. Lumps or clots of fat were not found and the fat separation did not reach an unacceptable level of 3 at any storage temperature during the 52 weeks of storage (Table 1).

**Table 1:** Summary of sensory evaluation measured during long-term storage of ultra-high temperature (UHT) treated milk at 4, 20, 30 and 37 °C. Values correspond to storage time in weeks when the UHT milk was considered no longer accepted by consumers. For sediment formation, there was a difference between UHT milk produced at the dairy plant and the pilot plant.

Sensory attribute	Definition	Scale	Value when no longer acceptable for consumption	Storage temperature			
				4 °C	20 °C	30 °C	37 °C
Taste	Perceived taste	1 = no deviation 2 = small deviation 3 = large deviation	≥2	48-52 w	40-44 w	24-32 w	24-32 w
Colour	Measured colour	L*, a* and b* values	L* values < 76 a* values > -1 b* values > 7	>52 w	>52 w	28-30 w	16-20 w
Fat separation	Perceived thickness of the fat layer floating on the surface	0 = no separation 1 = waves 2 = covered 3 = lumps/clots	3	>52 w	>52 w	>52 w	>52 w
Fat adhesion	Thickness and size of the fat sticking to the inside of the package, after the milk is poured out, compared with reference photos	0 = no fat layer 1 = small 2 = medium 3 = large 4 = large and thick	≥3.5	>52 w	>52 w	46 w	28 w
Sediment formation	Size of the sediment adhering to the bottom of the package, after the milk is poured out, compared with reference photos	0–100%	>45%	dairy plant 34 w pilot plant 40 w	36 w 52 w	20 w >52 w	24 w >52 w

Fat adhesion increased with storage temperature and time (Fig. 4B). UHT milk stored at 4 °C had least adhesion, on average evaluated as 2, on the scale 0–4, after 52 weeks of storage. At 20 °C, most samples had a fat adhesion corresponding to a 2 on the scale after 16–20 weeks of storage and at 40 weeks samples had on average a 3. At 30 and 37 °C fat adhesion corresponded to 2 after 6–14 weeks of storage, and at the end of storage fat adhesion in all samples were rated 3 or 4. In this study, values for fat adhesion ≥3.5 were regarded as not acceptable product quality. This level was never reached in UHT milk stored at 4 and 20 °C but was reached in UHT milk stored at 30 °C for 46 weeks or at 37 °C for 28 weeks.

UHT milk originating from the two production sites, commercial dairy plant and pilot plant, were similar with respect to changes in stability during storage except for the extent of sedimentation. More sediment was formed in UHT milk produced at the dairy plant, using raw milk, compared to the pilot plant, using pasteurised milk (Fig. 4C). For UHT milk stored at 4 °C produced at the dairy and pilot plant, formation of sediment was visual after 12 and 24 weeks of storage, respectively, covering the entire bottom of the packages (100%) after 52 weeks of storage (Fig. 4C). Initially, more sediment was formed at the higher storage temperatures. However, from storage week 40 (dairy plant) and 30 (pilot plant), sediment formation was highest in UHT milk stored at 4 °C compared to the other storage temperatures. In UHT milk stored at 20, 30 and 37 °C, from the onset of sedimentation there was a continuous increase and at the end of the storage period UHT milk produced at the dairy plant and pilot plant reached 70–80 and 30–40% sediment, respectively. Despite the long-term storage no age gelation was found.

The storage time when UHT milk was considered no longer acceptable for consumption based on different sensory attributes are found in Table 1. Based on the scales used in this study to evaluate sensory traits of the UHT milk, sedimentation covering >45 % of the bottom of the package limited the shelf-life of UHT milk stored at 4 °C to 34–40 weeks. For UHT milk produced at the dairy plant and stored at 20 °C the shelf-life was limited to 36–44 weeks by deviating taste and sediment formation. For UHT milk produced at the dairy and stored at 30 °C the shelf-life was limited to 20–32 weeks due to limitations in several quality parameters including sediment formation and a deviating taste, shortly followed by an unacceptable colour in packages from both production sites. During storage at 37 °C the shelf-life was limited to 16–20 weeks by changes in colour, closely followed by excess sediment and a deviating taste. In this study, fat separation was, probably due to the low fat content of 1.5%, not regarded as problematic from a consumer perspective.

### 3.2 Heat coagulation time, pH and ethanol stability

**Fig. 5:**
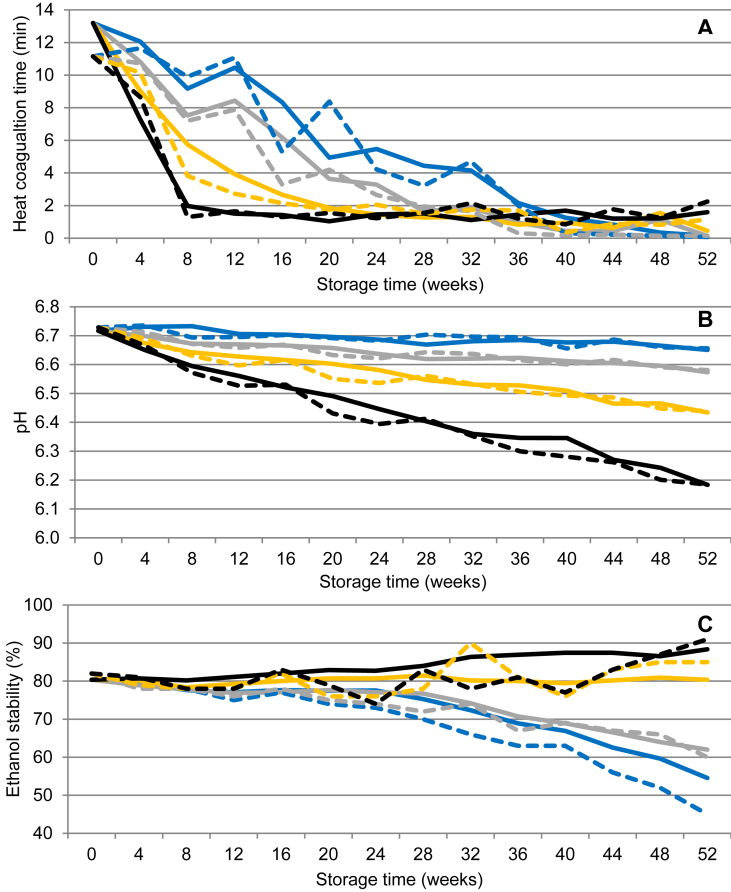
Changes in heat coagulation time, pH and ethanol stability in ultra-high temperature treated milk during storage from 0-52 weeks at 4 °C (blue), 20 °C (grey), 30 °C (yellow) and 37 °C (black). Values represent average values of eleven batches of UHT milk produced at a dairy plant (solid line) and two batches of UHT milk produced at a pilot plant (dashed line).

UHT milk had an initial HCT of 11–13 min (Fig. 5A). After 36 weeks of storage at 4 °C, HCT had decreased to <2 min, a more or less instantaneous coagulation. For UHT milk stored at 37 °C, HCT decreased to <2 min already after 8 weeks of storage.

The initial pH of 6.7 remained in UHT milk stored at 4 °C during the 52 weeks of storage (Fig. 5B). At the highest storage temperature (37 °C), there was more or less a linear decline in pH from storage week 0 to 52, with a final pH of 6.2.

The freshly produced UHT milk had an initial ethanol stability of >80%, during storage at 4 °C decreasing to 45–55%, and when stored at 20 °C to 60% after 52 weeks of storage (Fig. 5C). Interestingly, in UHT milk stored at 30 and 37 °C there was a tendency for increased ethanol stability during storage.

### 3.3 Correlation

Correlation coefficients for all investigated parameters were calculated (Table 2). In our study, for all storage temperatures, fat separation, fat adhesion and sediment formation were strongly positively correlated. At 30 and 37 °C, where we during storage observed changes in colour, L* values were strongly negatively correlated to a* and b*values and a* and b* values were positively correlated to each other. Further, at the warm storage temperatures, variations in colour were correlated to pH. For UHT milk stored at 4 and 20 °C sediment formation was negatively correlated to ethanol stability, at 30 °C there was no significant correlation and at 37 °C the correlation was positive. For all storage temperatures, sedimentation was significantly negatively correlated to heat coagulation time. Heat coagulation time and ethanol stability was positively correlated at 4 and 20 °C, but uncorrelated at 30 and 37 °C.

**Table 2:** Pearson correlation coefficients for ultra-high temperature treated milk produced at a commercial dairy plant, stored 0–52 weeks at 4, 20, 30 and 37 °C ***p ≤ 0.001, **p ≤ 0.01, *p ≤ 0.05 and n.s. not significant.

	L*	a*	b*	Fat separation	Fat adhesion	Sedimentation	Heat coagulation time	pH	Ethanol stability
**Storage temperature 4 °C**									
Taste	-0.195*	-0.270***	n.s.	0.303***	0.214**	0.243**	-0.255**	-0.237**	-0.185*
L*		0.369***	n.s.	-0.381***	-0.444***	-0.482***	0.405***	n.s.	0.451***
a*			0.279***	-0.241**	-0.356***	-0.323***	0.303***	0.196*	0.223**
b*				n.s.	n.s.	-0.163*	n.s.	n.s.	0.163*
Fat separation					0.688	0.736***	-0.674***	-0.419***	-0.686***
Fat adhesion						0.797***	-0.728***	-0.284***	-0.661***
Sedimentation							-0.785***	-0.398***	-0.859***
Heat coagulation time								0.427***	0.655***
pH									0.342***
**Storage temperature 20 °C**									
Taste	-0.302***	-0.227*	n.s.	0.343***	0.337***	0.379***	-0.278***	-0.288***	-0.390***
L*		0.375***	0.241**	-0.561***	-0.656***	-0.729***	0.656***	0.587***	0.589***
a*			0.708***	n.s.	-0.209**	n.s.	n.s.	n.s.	0.164*
b*				n.s.	-0.194*	n.s.	n.s.	n.s.	0.162*
Fat separation					0.761***	0.788***	-0.827***	-0.639***	-0.606***
Fat adhesion						0.815***	-0.812***	-0.673***	-0.589***
Sedimentation							-0.804***	-0.736***	-0.801***
Heat coagulation time								0.706***	0.603***
pH									0.646***
**Storage temperature 30 °C**									
Taste	-0.350***	n.s.	0.270***	0.352***	0.332***	0.447***	-0.312***	-0.429***	-0.264***
L*		-0.790***	-0.861***	-0.613***	-0.650***	-0.734***	0.556***	0.837***	-0.330***
a*			0.949***	0.434***	0.526***	0.528***	-0.398***	-0.684***	0.536***
b*				0.525***	0.595***	0.655***	-0.457***	-0.774***	0.435***
Fat separation					0.776***	0.815***	-0.832***	-0.705***	n.s.
Fat adhesion						0.862***	-0.807***	-0.765***	n.s.
Sedimentation							-0.802***	-0.838***	n.s.
Heat coagulation time								0.701***	n.s.
pH									n.s.
**Storage temperature 37 °C**									
Taste	-0.514***	0.452***	0.522***	0.344***	0.408***	0.408***	-0.258**	-0.505***	n.s.
L*		-0.879***	-0.928***	-0.654***	-0.759***	-0.775***	0.461***	0.875***	-0.605***
a*			0.927***	0.614***	0.764***	0.694***	-0.483***	-0.832***	0.634***
b*				0.727***	0.851***	0.841***	-0.529***	-0.934***	0.566***
Fat separation					0.826***	0.810***	-0.785***	-0.743***	0.323***
Fat adhesion						0.896***	-0.748***	-0.851***	0.420***
Sedimentation							-0.731***	-0.871***	0.373***
Heat coagulation time								0.607***	n.s.
pH									-0.454***

## 4 Discussion

### 4.1 Taste, colour, fat separation, fat adhesion, sediment and gelation

Our results confirm that refrigerated or ambient storage temperatures are conditions that give good sensory properties for UHT milk. Only small flavour deviations were found after long-term storage at 4 and 20 °C, whereas at the higher storage temperatures 30 and 37 °C the UHT milk developed taste deviations earlier. Mechanisms for formation of off-flavours during long-term storage are many and complex. In milk, the sweet taste originates from available sugars, e.g. lactose, galactose and glucose. Earlier studies by (Jensen et al. (2015) and Clare et al. (2005) observed a decline in sweetness during storage of UHT milk. It was suggested to be caused by a reduction in free sugars available, e.g. lactose, galactose and glucose, due to ongoing Maillard reactions. In contrast, we observed an increase in sweetness in some milk samples, suggesting that the formation of or decrease in levels of other taste active compounds, may also contribute to sweet flavour. A cardboard-like flavour could originate from the oxidation of phospholipids during the long-term storage (Walstra et al., 2006). The creamy and watery perception observed in our study has previously been described by Jensen et al. (2015) and is in their study suggested to depend on the distribution of fat and protein in milk. In agreement with the results from the warm storage temperatures, previous studies have shown that in heated dairy products off-flavours can originate from the Maillard reaction, resulting in brown coloured, caramel and acidic flavoured products (Clare et al., 2005).

In UHT milk stored at 30 and 37 °C, where we had changes in colour during storage, strong correlations between L*, a* and b* values were found (Table 2). In our study, the changes in colour (Fig. 3) correspond with Al-Saadi and Deeth (2008) who found that the Maillard reaction proceeds faster with increasing temperature, explaining difference in colour between storage temperatures. Our results agree with Auldist et al. (1996), reporting L* values of 77–78 after storage of UHT milk for 6 months at 20 °C. Measured b* values in UHT milk stored at 40 °C for up to 26 weeks was found to increase from 7.3 to 14.6 arbitrary units (Gaucher et al., 2008a,b). In a recent study, changes in colour were measured in skimmed UHT milk during storage at 10–50 °C for up to 24 weeks and, in agreement with findings in our study, L*, a* and b* values were found to change linearly with storage time (Sunds et al., 2018).

In this study, fat separation increased with storage time and storage temperature, but due to the low fat content of 1.5 % and efficient homogenisation, fat separation was not an issue and fat adhesion was only regarded as a minor problem after long-term storage at 30 and 37 °C (Table 1). Methods for measuring fat separation and adhesion vary, and so does the results from earlier studies. Despite an extensive fat rise in UHT milk stored at 15 °C for 20 weeks, Deutsch and Jackson (1970) only registered few complaints in their study, indicating a high consumer acceptance for fat separation. Hansen et al. (1980) observed no fat separation when UHT milk (3.25% fat) was stored at 4 °C for 24 weeks; in contrast, fat separation was regarded as problematic in UHT milk stored at 24 and 40 °C for 12 weeks. Mechanisms for fat separation and, to some part, fat adhesion can be explained by Stoke's law. As storage temperature increases, the viscosity decreases and at the same time differences in density between fat and liquid phase increase, allowing faster movement of fat globules, explaining the fat adhesion at the warm storage temperatures. The strong positive correlation between fat separation, fat adhesion and sediment formation for all storage temperatures (Table 2) support the suggestion that they can be explained by Stoke's law.

In our study, sediment formation was found already at 4–12 weeks of storage at 30 and 37 °C (Fig. 4C). The sediment formation was highest at the higher temperatures, and increased during storage, at all storage temperature. Our results are in agreement with the observations by Ramsey and Swartzel (1984), who stored UHT milk at 7, 22 and 35 °C for 26 weeks and found the amount of sediment formed to increase with storage temperature. Interestingly, in our study after 30–40 weeks of storage, the speed of sediment formation at cold storage exceeded that at higher temperatures (Fig. 4C) and at 52 weeks of storage, the highest amount of sediment, covering the entire bottom of the package, was found in UHT milk stored at 4 °C. Independent of storage temperature, it is suggested that sediment consists of destabilised κ-casein depleted micelles formed by the UHT process (Gaur et al., 2018; Malmgren et al., 2017). The result is the formation of larger heavy particles setting at the bottom following Stoke's law (Deeth and Lewis, 2016). Increased storage temperature is suggested to accelerate sedimentation, due to a lower viscosity of the milk serum (Anema, 2018), also explaining the sedimentation observed at higher storage temperatures in our study. Possible explanations for sediment formation after 30–40 weeks of storage at 4 °C are hard to find in the literature. The decrease in hydrophobic interactions (Walstra et al., 2006) and the solubilisation of calcium phosphate at low temperature (Pyne, 1962) will contribute to a solubilisation of caseins, which could lead to destabilisation during cold storage. It is also possible that what we see in our study is a transition from sedimentation to initial age gelation. At least two mechanisms are known to cause age gelation; enzymatic or non-enzymatic (Anema, 2018). Analysing enzymatic activity in our UHT milk from four production months from the commercial dairy plant, no plasmin activity could be detected during the 52 weeks of storage (data not shown). In the non-enzymatic mechanism, also called physico-chemical age gelation, proteins are not hydrolysed during storage (Anema, 2018). The physico-chemical process is slow, gelation is rarely observed within 12 months of storage (Anema, 2018) and it tends to be favoured by low storage temperature (Samel et al., 1971), in agreement with our results. The difference in sediment formation between the dairy plant and the pilot plant (Fig. 4C) could possibly be due to small differences in raw material and processing conditions e.g. differences in composition, age of the milk, enzymatic activity or heat load by using raw milk at the dairy plant and pasteurised milk at the pilot plant, respectively, for further UHT processing.

### 4.2 Heat coagulation time, pH and ethanol stability

Cold storage gave the most heat stable casein micelles, in our study observed as a slower decrease in HCT during storage at 4 °C (Fig. 5A). Already at 8 weeks of storage at 37 °C, the pH of our UHT milk had decreased below pH 6.6 (Fig. 5B). With pH decreasing during storage, electrostatic repulsion between casein micelles will be reduced (Walstra, 1990). Polymerization-induced coagulation of casein micelles will be favoured by pH < 6.7, resulting in a shorter HCT (Deeth and Lewis, 2016; van Boekel et al., 1989), and possibly contributing to the low heat stability in UHT milk stored at elevated temperature, in our study 30 and 37 °C. Al-Saadi and Deeth (2008), investigating changes in UHT milk during storage for 12 weeks at 5, 20, 37 and 45 °C, found that the initial pH of 6.61 decreased to 6.56, 6.50, 6.43 and 6.35 at the respective storage temperature. Hardham et al. (2000) observed that pH decreased by less than 0.1 units in UHT milk stored at 25 °C for 9 months, whereas Gaucher et al. (2008a,b) reported a decrease of 0.5 pH units when UHT milk was stored at 40 °C for 6 months. Changes in pH and HCT during storage are likely to be associated to the progress of the Maillard reaction especially at 30 and 37 °C. In this complex reaction, besides formation of brown pigments, lactose is subject to isomerization and degradation, creating significant amounts of formic acid, being largely responsible for the storage-induced decline in milk pH (van Boekel, 1998). The rate of the Maillard reaction increases with temperature, explaining the differences in pH between storage temperatures (Al-Saadi and Deeth, 2008). The decrease in pH can also been attributed to protein cross-linking reactions or dephosphorylation of caseins resulting in a release of protons, whereby the extent of protein cross-linking has been shown to increase at elevated temperatures (Al-Saadi and Deeth, 2008; Holland et al., 2011).

Measuring ethanol stability during storage, we observed that in UHT milk stored at 4 °C ethanol stability decreased without a corresponding reduction in pH (Fig. 5C). The strongest correlation between ethanol and pH was found for UHT milk stored at 20 °C (Table 2). At 30 °C no correlation was found between pH and ethanol stability (Table 2). Interestingly, during storage at 37 °C, ethanol stability remained high despite the decrease in pH. Hence, the effect of pH on ethanol stability observed in raw milk cannot be applied on stored UHT milk. In recent publications, the visual coagulation of milk failing the ethanol stability test, is believed to originate from the outer hairy κ-casein layer collapsing onto the casein micelle surface as well as changes to the internal structure of the micelle, causing aggregation of the micelles (Day et al., 2017; Horne, 2016). Holland et al. (2011), analysing changes in the protein profile of UHT milk stored for 2 months at 4 °C, found that the α_s1_-casein fraction remained intact during storage. In contrast, after storage at 28 and 40 °C for 2 months, intra-micellar cross-links between α_s1_-, α_s2_-and/or β-casein were observed and suggested to be formed by non-enzymatic deamidation (Holland et al., 2011). Deamidation of asparagine and glutamine, increasing the net negative charge of the proteins, increased with storage temperature and time (Holland et al., 2011). Intra-micellar cross-linking could also be formed as part of the Maillard reaction, increasing at higher temperatures (Al-Saadi and Deeth, 2008; Holland et al., 2011). In this study, intra-micellar crosslinks may have contributed to maintaining a high ethanol stability in UHT milk stored at 30 and 37 °C due to stronger electrostatic repulsion between micelles (Horne, 2016).

## 5 Conclusion

In our study, the stability of UHT milk during storage was in general not affected by production scale (commercial dairy plant or pilot plant). Storage temperature was found to have a major impact on the stability of UHT treated milk during the 52 weeks of storage. A long shelf-life of UHT milk was favoured by cold or ambient storage temperatures, whereas the shelf-life decreased considerably when storage temperature increased. The shelf-life of UHT milk stored at 4 and 20 °C was limited by sediment formation to 34–36 weeks followed by a taste deviation at 40–52 weeks of storage. The shelf-life of UHT milk stored at 30 and 37 °C was limited by several quality parameters including taste, colour and sediment formation, from storage weeks 16–20. Changes in HCT, pH and ethanol stability of the UHT milk suggested that different mechanisms are attributed to the changes in stability at different storage temperatures.

## Declarations

### Author contribution statement

Maria A. Karlsson, Maud Langton, Fredrik Innings, Bozena Malmgren, Annika Höjer, Malin Wikström, Åse Lundh: Conceived and designed the experiments; Performed the experiments; Analyzed and interpreted the data; Contributed reagents, materials, analysis tools or data; Wrote the paper.

### Funding statement

This research did not receive any specific grant from funding agencies in the public, commercial, or not-for-profit sectors.

### Competing interest statement

The authors declare no conflict of interest.

### Additional information

No additional information is available for this paper.
